# 3-Dimensional coculture of breast cancer cell lines with adipose tissue–Derived stem cells reveals the efficiency of oncolytic Newcastle disease virus infection *via* labeling technology

**DOI:** 10.3389/fmolb.2022.754100

**Published:** 2022-09-12

**Authors:** Marwa Ibrahim Salman, Ahmed Majeed Al-Shammari, Mahfodha Abbas Emran

**Affiliations:** ^1^ Department of Biotechnology, College of Science, University of Baghdad, Baghdad, Iraq; ^2^ Department of Experimental Therapy, Iraqi Center for Cancer and Medical Genetic Research, Mustansiriyah University, Baghdad, Iraq

**Keywords:** labeled viruses, virotherapy, breast cancer, adipose tissue–derived adult stem cell, 3D cancer model, coculture

## Abstract

Oncolytic virotherapy is one of the emerging biological therapeutics that needs a more efficient *in vitro* tumor model to overcome the two-dimensional (2D) monolayer tumor cell culture model’s inability to maintain tissue-specific structure. This is to offer significant prognostic preclinical assessment findings. One of the best models that can mimic the *in vivo* model *in vitro* are the three-dimensional (3D) tumor–normal cell coculture systems, which can be employed in preclinical oncolytic virus therapeutics. Thus, we developed our 3D coculture system *in vitro* using two types of breast cancer cell lines showing different receptor statuses cocultured with adipose tissue–derived mesenchymal stem cells. The cells were cultured in a floater tissue culture plate to allow spheroids formation, and then the spheroids were collected and transferred to a scaffold spheroids dish. These 3D culture systems were used to evaluate oncolytic Newcastle disease virus AMHA1 strain infectivity and antitumor activity using a tracking system of the Newcastle disease virus (NDV) labeled with fluorescent PKH67 linker to follow the virus entry into target cells. This provides evidence that the NDV AMHA1 strain is an efficient oncolytic agent. The fluorescently detected virus particles showed high intensity in both coculture spheres. Strategies for chemically introducing fluorescent dyes into NDV particles extract quantitative information from the infected cancer models. In conclusion, the results indicate that the NDV AMHA1 strain efficiently replicates and induces an antitumor effect in cancer–normal 3D coculture systems, indicating efficient clinical outcomes.

## Introduction

Oncolytic virotherapy is an emerging biological therapeutic that needs an efficient *in vitro* tumor model to maintain the tissue-specific structure that two-dimensional (2D) culture models cannot maintain (Kloker et al., 2018; Duval et al., 2017). One of the best models that can mimic the *in vivo* model *in vitro* are the three-dimensional (3D) tumor–normal cell coculture systems (Chaicharoenaudomrung et al., 2019), which can be employed in preclinical oncolytic virus therapeutics. Oncolytic virotherapy is a kind of promising breast cancer therapy (Al-Ziaydi et al., 2020b). Breast cancer (BC) is the most invasive malignancy and the main cause of death in females (Cao et al., 2020). One of the most significant extrinsic variables in the development of BC is the tumor microenvironment (TME), which is composed of extracellular matrix (ECM) proteins, stromal cells such as adipocytes and associated cells, and the physical characteristics of neighboring cells or the ECM, all making up the TME structure (Place et al., 2011). These variables may have a cumulative effect against the development and progression of the tumor by influencing BC cell behavior *via* biophysical or biochemical interactions (Pallegar et al., 2019). Therefore, the 3D breast cancer cell systems can be used as a model that holds an inordinate promise for the discovery of drugs and cancer-targeted therapy. The NDV has been suggested as a biological agent with the potential to break therapy resistance, as it can replicate in non-proliferating tumor cells that are resistant to chemotherapy and radiotherapy (Schirrmacher, 2015). The NDV can also interfere with cancer angiogenesis (Al-Shammari et al., 2020a). The induction of apoptosis and immunogenic cell death is involved in NDV-mediated cancer cell killing (Al-Shammari et al., 2020c). Several methods have been developed to study virus adsorption and internalization, such as fluorescent *in situ* hybridization (Brabec-Zaruba et al., 2009), signal molecule tracking (Seisenberger et al., 2001), and radioactive labeling (Gotoh et al., 2006). Fluorescent lipid molecule tracing using amphipathic carbocyanine probe techniques has also been reported for labeling and visualizing enveloped RNA virus–cell interactions (Balogh et al., 2011). Here, we aim to demonstrate the efficiency of the NDV AMHA1 strain labeled with the PKH67 linker in replicating the 3D coculture spheroid cells augmented with scaffold developed to mimic the *in vivo* interaction between the BC cells and human adipose tissue–derived mesenchymal stem cells (hATMSCs).

## Material and method

The study was approved by the Scientific Committee of the Department of Biotechnology, College of Science, Baghdad University. The experiments were performed at the Experimental Therapy Department, Iraqi Center of Cancer and Medical Genetics Research (ICCMGR), Mustansiriyah University, Baghdad, Iraq.

### Newcastle disease virus propagation

An attenuated NDV AMHA1 strain (Al-Ziaydi et al., 2020a) was propagated in a 9-day chicken egg embryo (Al-Kindi, Baghdad, Iraq). The NDV was collected from the allantoic fluid of chicken eggs, purified from debris by using centrifugation at 3,000 rpm for 30 min, and stored at −80°C after being tested for hemagglutination (HA test). The viral titers were determined on Vero–SLAM cells (kindly provided by Dr. S.J. Russell, Mayo Clinic, United States) using a 50% tissue culture infective dose titration assay (TCID 50) per the standard procedure.

### Newcastle disease virus purification

The virus was purified from the allantoic fluids through the sucrose gradient method. First, the debris-free NDV was concentrated by ultracentrifugation at 120,000 *g* in an SW 32 Ti rotor (Beckman, United States) for 3.5 h at 4°C to make viral pellets suspended in 1 ml phosphate-buffered saline (PBS) of each tube and collected and stored at −80°C. The sucrose gradient was prepared as 60%, 50%, 40%, and 25% w/v sucrose in Milli-Q water, 0.22 mM filter sterilized, in 13.2 ml ultracentrifuge tubes at 4°C. The gradient was prepared by adding 1 ml of 60% w/v sucrose to the bottom of the tube. Later, it was carefully layered with 2 ml, 2 ml, and 2.5 ml of 50%, 40%, and 25% w/v sucrose, respectively. Finally, a layer of 4 ml of concentrated NDV was added to the top. The gradients were ultracentrifuged at 120,000 *g* in an SW 41 Ti rotor for 3.5 h at 4°C. The virus typically bands between the 40% and 50% sucrose layers. All tubes had to be clamped to retort stands to collect the virus, then wiped with 70% ethanol from the outside. A wide-mouthed container was placed below the ultracentrifuge tube to collect the liquid waste that was poured out after removing the syringe. An 18-G needle was attached to a 3 cc syringe positioned to the side of the tube at around 5 mm below the virus-containing band and the virus (2 ml) was slowly withdrawn (Santry et al., 2018).

### Cancer and normal cells

The human breast cancer cell line AMJ13 derived from Iraqi patients (estrogen and progesterone receptors negative) (Al-Shammari et al., 2015), the MCF7 human BC cell line (estrogen, progesterone receptors positive), and normal human adipose tissue–derived mesenchymal stem cells (hATMSCs) were supplied as a cell line established by Dr. Ahmed Majeed Al-Shammari, Experimental Therapy department, the Iraqi Center of Cancer and Medical Genetics Research (ICCMGR), Mustansiriyah University, Baghdad, Iraq. The original hATMSCs sample isolation was described by Hammadi and Alhimyari (2019). AMJ13 was cultured in an RPMI-1640 medium. The MCF7 cell and hATMSCs were cultured in MEM (US Biological, United States) supplemented with 10% (v/v) fetal bovine serum (FBS) and 100 IU penicillin and 100 µg streptomycin (Capricorn-Scientific, Germany). The cells were checked regularly for contamination.

### Three-dimensional multicellular spheroids coculture system

We modified the 3D-culture protocol initially developed by Wong et al. (2012) and Rolver et al. (2019). Briefly, Both AMJ13 cancer cells and hATMSCs normal cells, MCF-7, and hATMSCs, were trypsinized from a monolayer to a single-cell suspension and seeded as coculture at 50,000 cells each (ratio: 1:1) in a 24-well cell floater plate (SPL3D™, SPL Life Sciences, South Korea) and allowed for spheroids formation for 3 days at 37°C. Later, we collected spheroids and transferred them to a scaffold spheroids dish for culturing for an additional 72 h, as the meshes inside the well facilitate identifying and counting the spheroids (mesh thickness: 137 µm, pore size: 200 µm, cat. No. 110350, SPL Life Sciences, South Korea).

### Newcastle disease virus particles' labeling with PKH67 linker

The PKH67 Fluorescent Cell Linker Kits (Sigma-Aldrich, United States) were used to label the oncolytic NDV AMHA1. A total of 1.5 × 10^8^ particles in PBS were labeled as follows: 1 µl of PKH67 dye (Sigma, St. Louis, MO) was dissolved in 2 ml of Diluent C before labeling as per the manufacturer's recommendations (Sigma, St. Louis, MO). Two volumes of diluted PKH67 were mixed with one volume of NDV suspension by pipetting. After 30 s, the labeling reaction was stopped by adding three volumes of the full medium and pipetting the suspension 5–6 times. The labeled virus was ready for exposure (Balogh et al., 2011).

### Coculture spheroids infection

Coculture spheroids were treated with labeled NDV at a multiplicity of infection (MOI) of 10 for 24 h before ending the experiment. Another group of spheroids was treated with NDV AMHA1 without adding PKH67 Fluorescent Linker to act as the non-labeled control. The PKH67–Diluent C mixture was incubated with ×1 PBS as described for virus labeling (without adding NDV) to prepare the mock-infected control. The reaction was halted by adding the full medium, and the mixture was then used to treat the cells. After 24 h, we aspirated the medium from the spheres and rinsed them twice with ice-cold PBS. We then fixed the culture with 4% paraformaldehyde at room temperature for 10 min and stopped the fixation process by aspirating 4% paraformaldehyde and injecting at least two medium-volume PBS for 10 min. The samples were incubated overnight at 4°C before removing paraformaldehyde to stain the cell nuclei with propidium iodide dye (PI) (10 μl PI + 1 ml PBS) (Balogh et al., 2011).

### Two-dimensional coculture protocol

In an 8-well chamber slide (SPL Life Sciences, South Korea), we seeded 2,500 cells of AMJ13 or MCF7 breast cancer cells with 25,000 normal hATMSCs in 400 µl of the growth media to allow cell growth overnight to create the 2D coculture system of AMJ13 with hATMSCs and MCF7 with hATMSCs. By day 2, the cells were treated with an NDV-PKH67 to detect virus infectivity and selectivity in the 2D coculture system.

### Image quantitative analysis

Images were analyzed using the ImageJ software to measure the green fluorescent dye and the sphere’s area. Moreover, the fields were quantified for sphere numbers. The growth inhibition of the spheres was assessed using the following formula:

$$Growth\;inhibition\;\%=\left(spheres\;{area}_{control}-spheres\;{area}_{treated}\right)/spheres\;{area}_{control}\times 100$$ (1)

where the spheres area_control_ is the mean sphere area of untreated wells, and spheres area_treated_ is the sphere area of treated wells.

### Investigation of Newcastle disease virus AMHA1 hemagglutinin–neuraminidase protein expression in cancer and normal spheroids to confirm virus replication in three-dimensional systems

The cancer spheroids (AMJ13 and MCF7) and normal hATMSCs spheroids were grown separately and infected with NDV at an MOI of 10 for 24 h to confirm infectivity and replication according to cell identity. These spheroids were washed with PBS, then fixed using 4% paraformaldehyde for 30 min at RT. Permeabilization was done using 0.5% Triton-X for 30 min, RT. They were blocked by using 10% goat serum for 60 min. The spheroids were stained with 1 µg/100 µl of the mouse monoclonal antibody raised against hemagglutinin–neuraminidase (HN) of the Newcastle disease virus [NDV-HN (11F12):sc-52112: dilution 1:30, Santa Cruz Biotechnology, CA, United States], diluted in blocking buffer, and incubated overnight at 4°C. Then, the spheroids were treated with 1 µg/100 µl of the secondary antibody Alexa Fluor 568–conjugated goat anti-mouse IgG for 2 h at RT. The spheroids were examined using a Micros fluorescent microscope and photographed using a Micros 5-megapixel camera (Micros, Austria). The fluorescence intensities were measured using the ImageJ software.

### Statistical analysis

Statistical significance was determined by unpaired two-tailed Student’s *t*-test or two-way analysis of variance with multiple comparisons using GraphPad Prism 6 (GraphPad Software, San Diego, CA). The statistical significance was set at *p* < 0.05.

## Result

### Spheres formation

**FIGURE 1:**
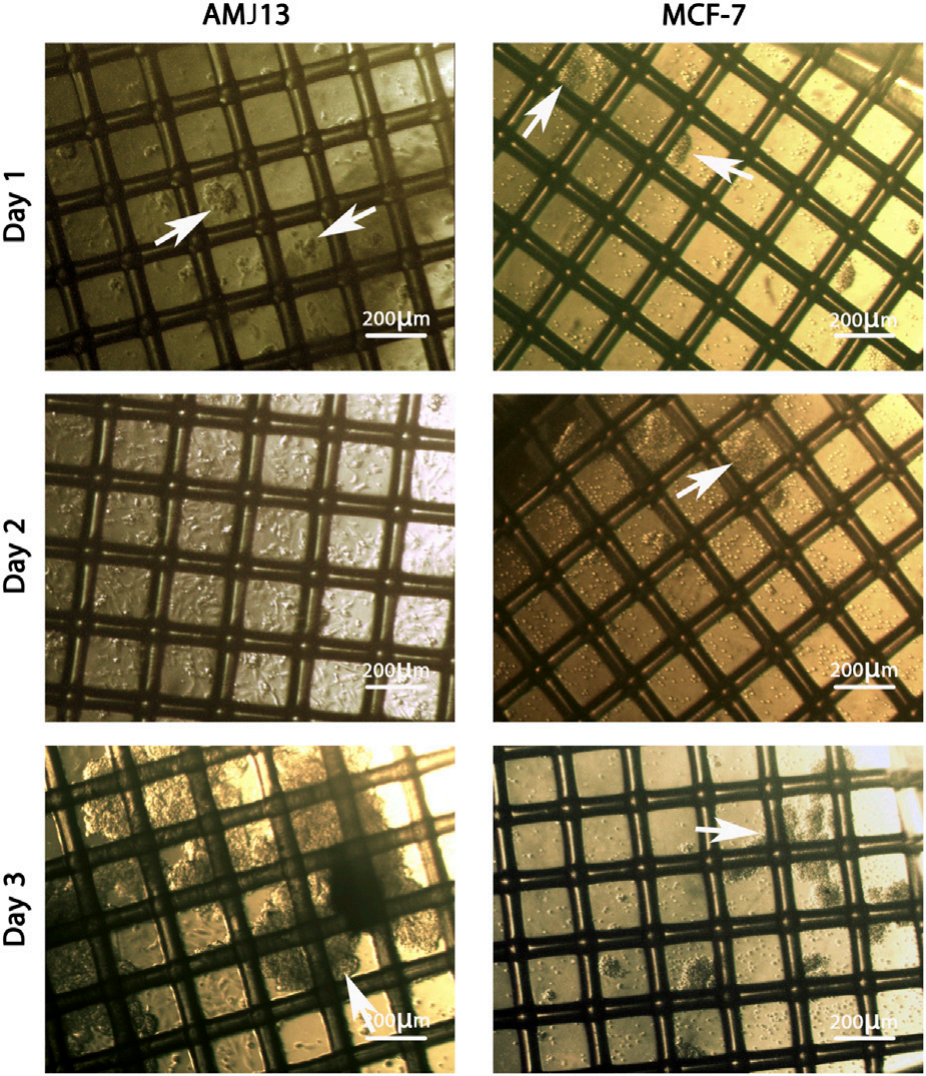
The growth of the spheroids coculture system of AMJ13-hATMSCs and MCF7-hATMSCs on a scaffold dish under an inverted microscope. On the first day, the spheres were minor in size, and the tiny aggregates of spheres (white arrows) were organized as a small network. At 48 h, the spheres contained a small number of cells individually, while another aggregate had many cells aggregated (white arrows). At 72 h, the spheres of the coculture extend in size to connect with the scaffold fiber and form massive spheroids (white arrows).

The 3D coculture spheres showed steady expansion and growth in the mesh scaffold. In general, during the first 24 hours, the aggregates of spheres were very tiny in size and less in number, which floated in the culture media (Figure 1). During the second day, the spheres had developed in size and increased in numbers. However, such spheres contained a small number of cells individually, while another aggregate had many cells aggregated. Nonetheless, during the third day, we observed hyperactive growth of spheres with a layer of huge aggregate on the surface of the suspended media (Figure 1).

### PKH67-labeled Newcastle disease virus AMHA1 lyses breast cancer–hATMSCs spheroid cocultures

**FIGURE 2:**
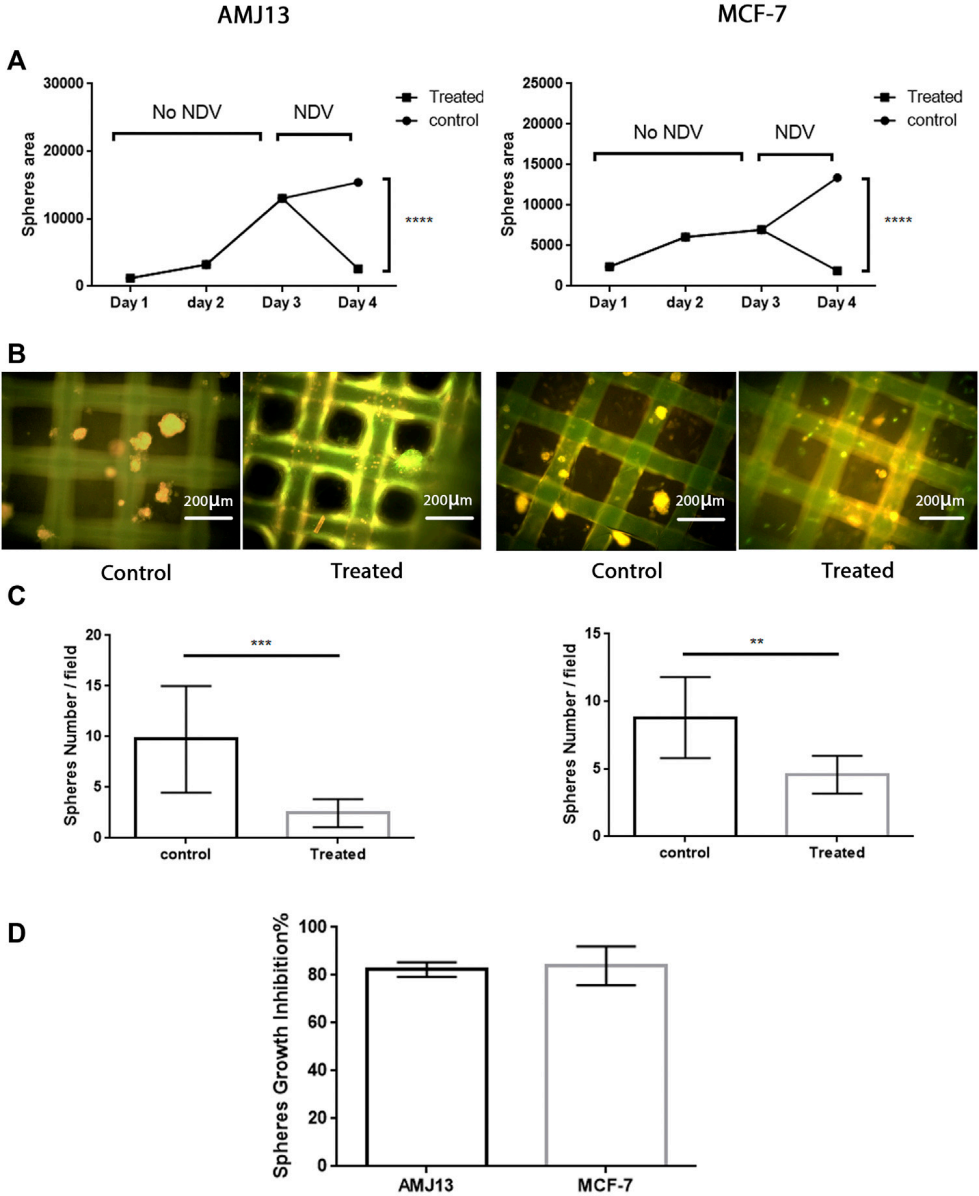
Antitumor assessment of the PKH67-NDV AMHA1 against the 3D breast cancer–hATMSCs spheres. Three-dimensional cocultures of AMJ13 and MCF7 cell lines and normal stem cells were exposed to labeled NDV AMHA1 at an MOI of 10 for 24 h post-infection. **(A)** Growth curve showing the steady growth of the spheres in size as measured by area, through 3 days until exposed to PKH67-NDV, where the spheres size drops due to oncolysis effect when compared to that of mock-infected cells. **(B)** Immunofluorescence image showing mostly green fluorescent PKH67-NDV infection decreased the number of spheres when compared to the control noninfected spheres. The control spheres show orange propidium iodide that stains the cell nuclei red. **(C)** Significant decreases seen in spheres number. **(D)** Sphere’s growth inhibition showed more than 80% for each cell line tested, indicating that PKH67-NDV can efficiently prevent breast cancer spheroids’ growth.

We first assessed the efficiency of PKH67-labeled NDV (PKH67-NDV) AMHA1 to revoke the 3D breast cancer–hATMSCs growth. Three-dimensional cocultures of AMJ13 and MCF7 cell lines were exposed to the labeled NDV AMHA1 at an MOI of 10 or mock-infected (control) and checked for spheroid size and numbers at 24 h post-infection. PKH67-NDV infected AMJ13, and MCF7 spheres were lysed or reduced in size when compared to mock-infected cells for the first 3 days before infection (Figures 2A,B). Likewise, PKH67-NDV infection significantly decreased the sphere numbers (Figure 2C). Moreover, the growth inhibition of the spheres show that more than 80% for each cell line has been tested (Figure 2D), indicating that PKH67-NDV can efficiently prevent breast cancer spheroids’ growth.

### Efficient infectivity of PKH67-labeled Newcastle disease virus in AMJ13 and MCF7 breast cancer three-dimensional spheroids

**FIGURE 3:**
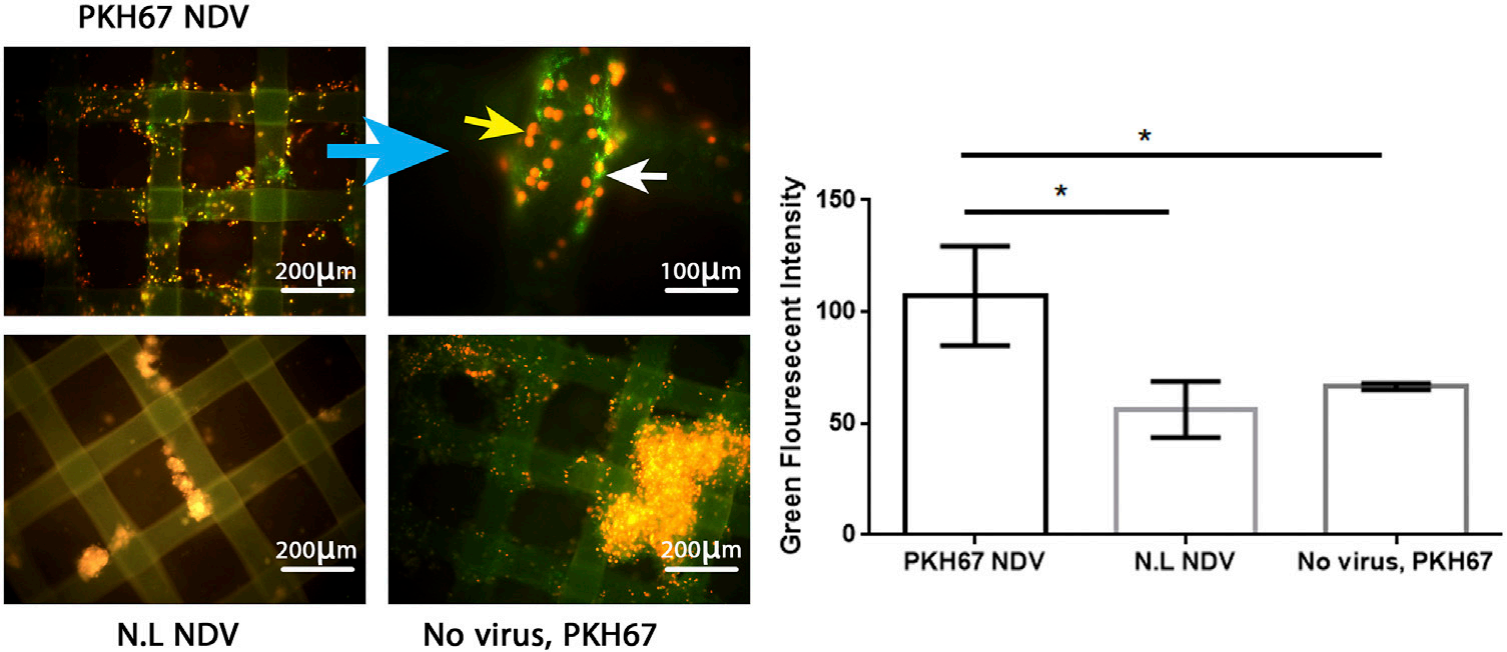
Infectivity analysis of PKH67-NDV in AMJ13-hATMSCs 3D spheroids. In representative fluorescent images of the 3D coculture of AMJ13-hATMSCs in the (3D) scaffold system, spheroids were infected at an MOI of 10 in the image labeled with PKH67-NDV. Cells were infected with labeled NDV (virus-carrying PKH67 linker). At 24 h post-infection, coculture spheroids were fixed and propidium iodide (PI)–stained for nuclear staining. PI staining is seen as red/orange, while the cytoplasm is seen as clear green fluorescence, ensuring the entry of the labeled NDV. The top left figure shows images of AMJ13 coculture (×100), and the top right figure shows the magnified PKH67-NDV–infected spheroids (×400). While the figure on the bottom left shows an image of non-labeled NDV infecting the 3D coculture system, showing lysed and small spheroids stained only with red-orange propidium iodide dye. The picture at the bottom right presents coculture spheroids of the AMJ13 spheroid cells treated with PKH67 linker only without any virus (mock-infected) as the second control. While the histogram clarifies the intensity of PKH67 green fluorescence, revealing that PKH67-NDV–infected spheres are the ones showing the most intense green fluorescence.

**FIGURE 4:**
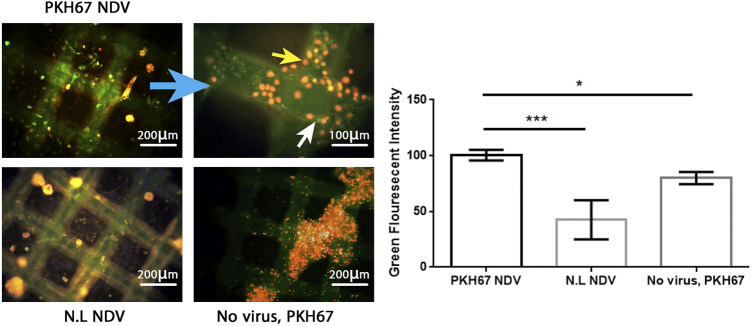
Infectivity analysis of PKH67-NDV in MCF7-hATMSCs 3D spheroids, coculture images of MCF7 with hATMSCs in the (3D) scaffold system; spheroids are infected at an MOI of 10 as seen under a fluorescence microscope. Cells were infected with labeled NDV (virus carrying PKH67 linker). At 24 h post-infection, coculture spheroids cells were fixed and stained with propidium iodide (PI) for nuclear staining. PI staining is shown in red/orange, while the cytoplasm is shown in green fluorescence for infectivity of the PKH67-NDV inside cancer cells' cytoplasm, which confirms the entry of labeled NDV. Also seen are few fluorescent red-orange spheres with no green fluorescence, which can be explained as a sphere of normal hATMSCs only spared by the NDV as it is not replicated in the normal cells. The top left figure shows images of MCF7 coculture ×100, and the top right figure is of a higher magnification of PKH67-NDV–infected spheres, which appear destroyed, and shows the presence of cells with green cytoplasm (white arrow) and red-orange nuclei (yellow arrow) (×400). While the bottom left figure (non-labeled NDV) shows MCF7-hATMSCs coculture spheres treated with non-labeled NDV, having a nucleus stained red-orange with propidium iodide dye. The bottom right figure presents cocultured spheroids of MCF7 spheroids cells, treated with PKH67 linker only without any virus (mock-infected), treated as the second control. The histogram clarifies the intensity of PKH67 green fluorescence, demonstrating that PKH67-NDV–infected spheres have the highest intensity while showing high infection of the labeled virus.

To evaluate the efficacy of the oncolytic NDV AMHA1 strain, we employed PKH67 dye to label the virus. Through green fluorescence intensity analysis, we determined both AMJ13-hATMSCs and MCF7-hATMSCs 3D cancer cell spheroids susceptibility to PKH67-NDV (Figures 3, 4). The PKH67-NDV replicated within 24 h, infecting most of the spheroids, which when infected with an MOI of 10, dyed all cancer spheres with clear green fluorescence. Unlike untreated cells that fluorescent red-orange only for the propidium iodide that stains nuclei of live cells, prove the specificity of our labeling dye as a tracking system for the labeled virus. By examining under a fluorescent microscope (Macros, Austria), the infected 3D spheres revealed lytic cytopathic effects and robust green fluorescent signaling (Figures 3, 4). The PKH67-NDV tracking system results show that both AMJ13 and MCF7 cancer spheroids are greatly sensitive to PKH67-NDV infectivity, confirming the broad-spectrum antitumor nature of the oncolytic NDV. The efficient infectivity subsequently causes cancer cell death and virus progeny release. Moreover, the images of PKH67-NDV–infected spheres show a few spheres that are fluorescent red-orange only with no green fluorescence, which can be explained as a sphere of normal hATMSCs only spared by the NDV, as they do not replicate in normal cells.

### Newcastle disease virus infects and replicates in cancer cells while infecting but not replicating in normal hATMSCs in a two-dimensional system

**FIGURE 5:**
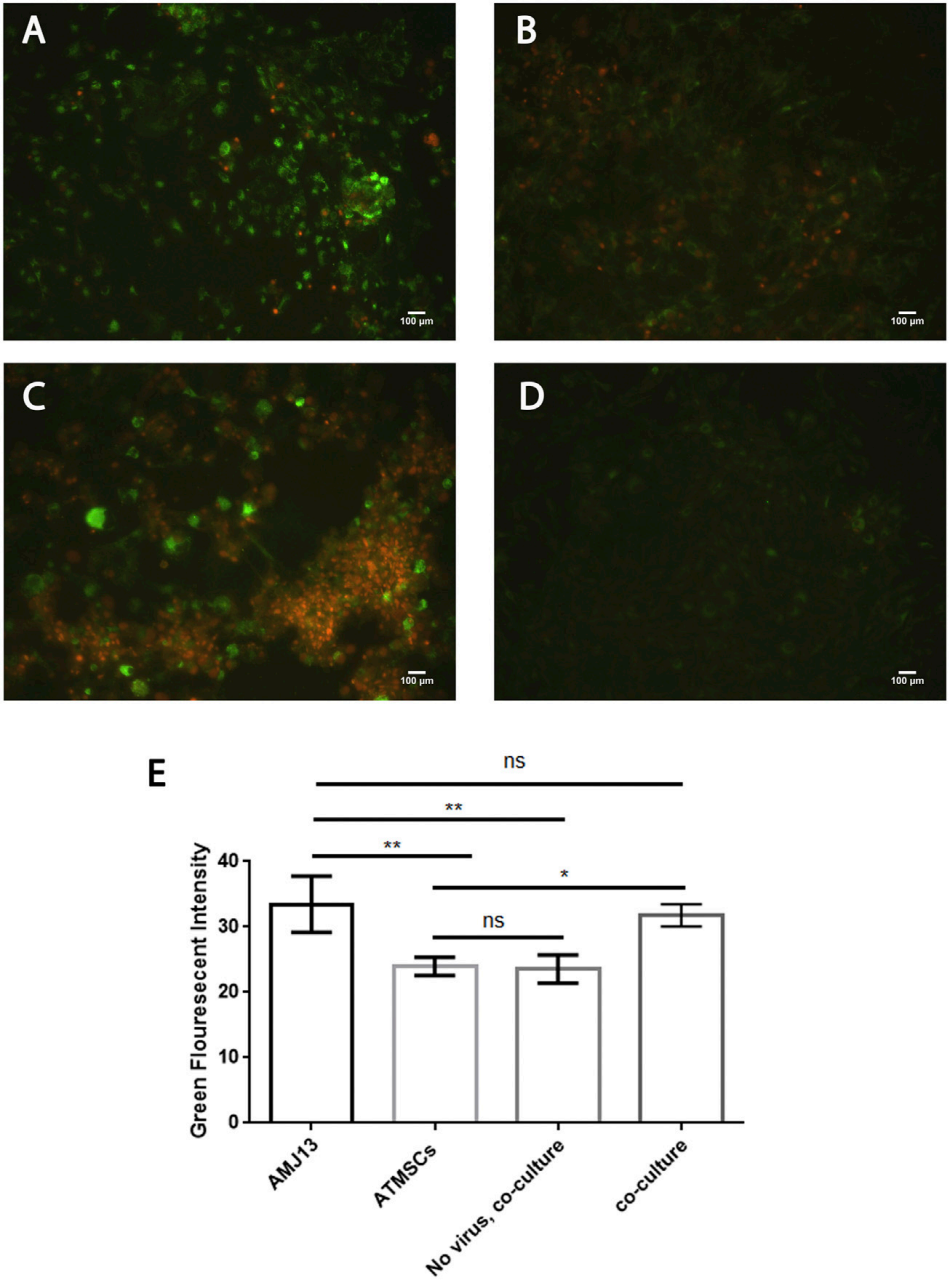
Newcastle disease virus infects and replicates in AMJ13 breast cancer cells while infecting but not replicating in normal hATMSCs. **(A)** AMJ13 breast cancer cells infected with labeled NDV. **(B)** Human normal stem cells infected with labeled NDV. **(C)** Coculture of AMJ13 cancer cells with human normal stem cells infected with labeled NDV. **(D)** Coculture model stained with PHK67 dye showing hardly detectable green fluorescence. **(E)** The quantitative image analysis for green fluorescence by ImageJ confirmed significant green fluorescence intensity of infected cancer cells when compared to normal infected cells and the noninfected coculture cell model. There was no significant difference in green fluorescence intensity seen between the infected cancer cells and infected cocultured cells. Propidium iodide was used as the counterstain.

**FIGURE 6:**
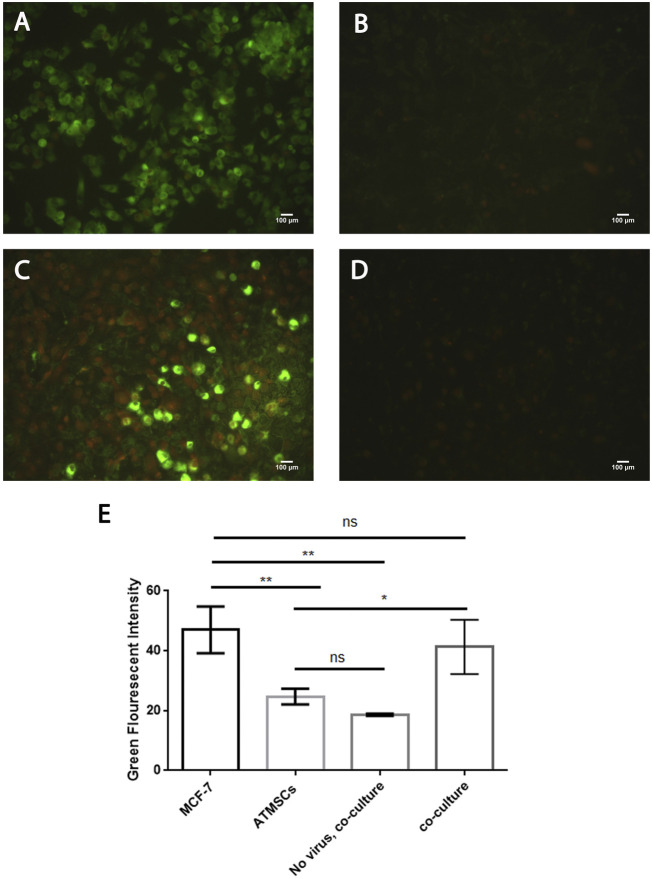
Newcastle disease virus infects and replicates in MCF7 breast cancer cells while infecting but not replicating in normal hATMSCs. **(A)** MCF7 breast cancer cells infected with labeled NDV. **(B)** Human normal stem cells infected with labeled NDV. **(C)** Coculture of MCF7 cancer cells with normal human stem cells infected with labeled NDV. **(D)** Coculture model stained with PHK67 dye showing hardly detectable green fluorescence. **(E)** The quantitative image analysis for green fluorescence by ImageJ confirmed significant green fluorescence intensity of the infected cancer cells as compared to normal infected cells and the noninfected coculture cell model. There was no significant difference in the green fluorescence intensity seen between the infected cancer cells and the infected cocultured cells. Propidium iodide was used as the counterstain.

We tested the hypothesis of NDV infection and replication in cancer cells while infecting but not replicating it in normal stem cells in a 2D cell culture model of cancer, hATMSCs, and coculture of both. We simulated the 3D experiments in a 2D model by culturing AMJ13 and MCF7 cancer cells with human normal stem cells and infecting them with labeled NDV, while adding the labeling dye to the cells as control. The results showed that both cancer cell lines (AMJ13 and MCF7) showed intense green fluorescent staining (Figures 5A, 6A), which reflects efficient NDV infection and replication in cancer cells as compared to normal cells that have very low green fluorescent intensity which is hardly detectable (Figures 5B, 6B). The 2D coculture model shows intense staining of some of the cells while others show only counterstaining (Figures 5C, 6C) after infection with PHK67-labeled NDV. Adding the PHK67 dye to the coculture model results in hardly detectable green fluorescence, which is even less than that seen in infected hATMSCs (Figures 5D, 6D). The quantitative image analysis for green fluorescence by ImageJ confirmed significant green fluorescence intensity of the infected cancer cells when compared to normal infected cells and the noninfected coculture cell model (Figure 5E, 6E). There was no significant difference in green fluorescence intensity between the infected cancer cells and infected cocultured cells. These data suggest that labeled viruses attached or entered normal cells and transferred their PHK67 to the cells without replicating in them while showing efficient infection and presence inside cancer cells.

### Expression of Newcastle disease virus AMHA1 hemagglutinin–neuraminidase protein in cancer spheroids but not in normal spheroids to confirm virus replication in three-dimensional systems according to cell identity

**FIGURE 7:**
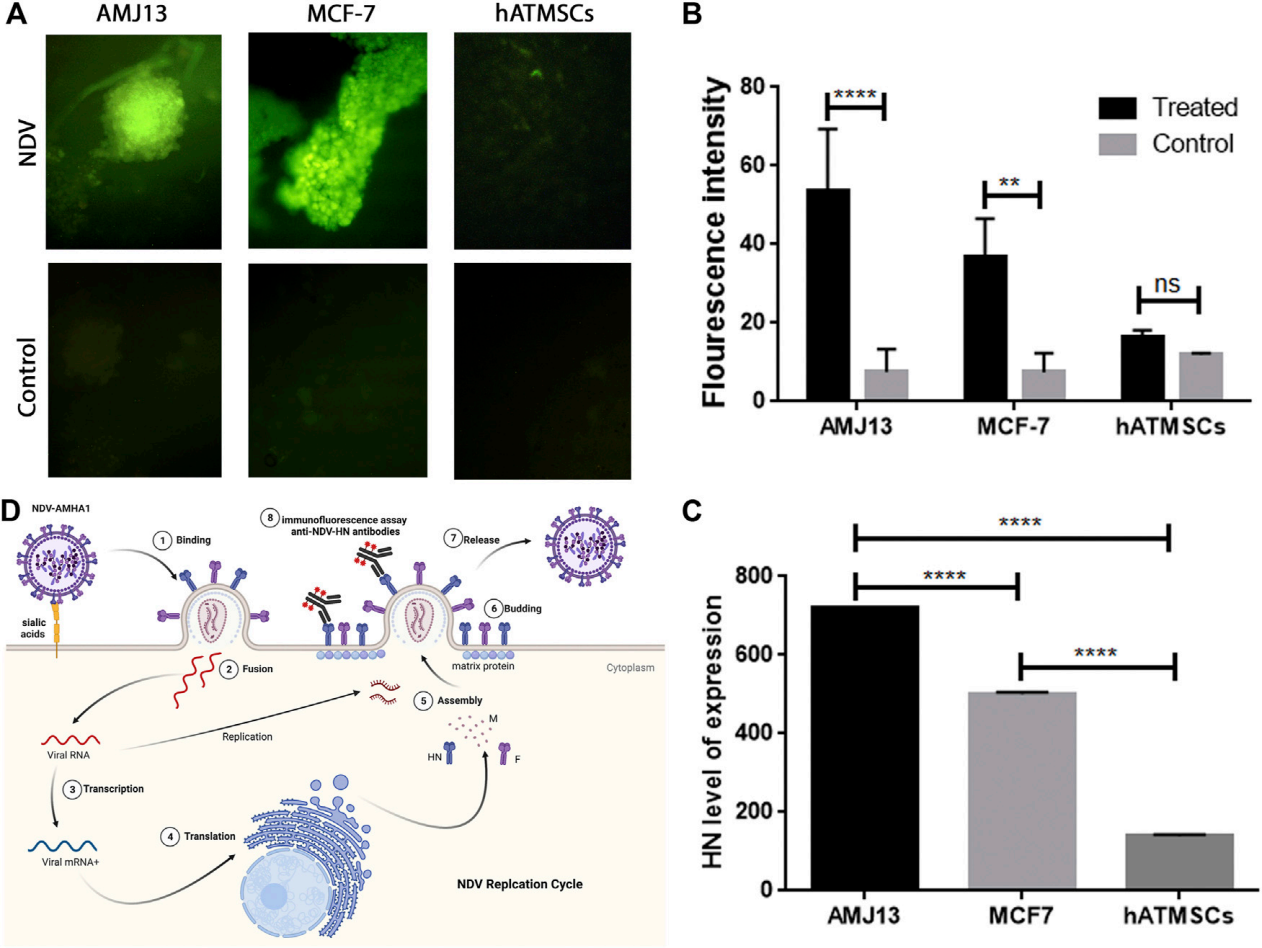
Immunofluorescence (IF) assay for NDV-HN protein detection confirms NDV AMHA1 infectivity and replication inside the spheroids through staining with anti-HN NDV mAbs. **(A)** Comparison between infected cells and the controls of all three spheroid types. Infected cancer spheroids expressing HN protein heavily, whereas there is no expression seen in the control or noninfected spheroids, while normal hATMSCs spheroids show low signal levels. **(B)** The fluorescence intensity analysis for the expression of HN-NDV protein in NDV-infected cancer and normal spheroids and comparison of the levels of expression showing significant HN protein expression levels in infected cancer spheroids when compared to noninfected cancer spheroids. Also, there were no significant HN protein levels in the infected normal cell spheroids when compared to noninfected normal spheroids. **(C)** Our findings show significant expression levels of HN-NDV in cancer cell spheroids that are NDV treated when compared to the untreated control cancer spheroids. Moreover, there is a significantly higher expression of HN-NDV in cancer spheroids (AMJ13 and MCF7) when compared to normal cells (hATMSCs). These results have confirmed that NDV AMHA1 could infect and replicate efficiently in cancer spheroids only with no significant replication in normal spheroids. **(D)** NDV AMHA1 replication cycle shows that HN protein is an important NDV protein inserted in the infected cell surface during the NDV replication cycle inside the cells. HN-NDV presence on the cell surface proves virus replication and infectivity.

Immunofluorescence (IF) assay for NDV-HN protein detection was performed to prove NDV infectivity and replication inside the spheroids by staining them with anti-HN NDV mAbs. HN is one of the main and important NDV proteins inserted in the infected cell surface during the NDV replication cycle inside the cells. Moreover, its presence in the cell is a great proof of virus replication and infectivity. Cancer and normal cell spheroids were grown separately to prove infectivity and replication according to cell identity. Figure 7A compares the infected cells and controls for all three spheroid types. Infected cancer spheroids express HN protein heavily, while there is no expression on the noninfected control spheroids and the normal hATMSCs spheroids show low signal levels. The fluorescence intensity analysis for the expression of HN-NDV protein in NDV-infected cancer and normal spheroids and comparison of the levels of expression showing significant HN protein expression levels in infected cancer spheroids compared to noninfected cancer spheroids. Also, there were no significant HN protein levels in the infected normal cell spheroids when compared to noninfected normal spheroids (Figure 7B).

We analyzed the expression of HN-NDV in NDV-infected cancer and normal spheroids and compared the levels of expression to prove NDV replication according to cell identity by replicating in cancer spheroids and sparing normal spheroids. Our finding was that there were significant expression levels of HN-NDV in infected AMJ13 cancer cell spheroids as compared to MCF7 cancer spheroids. Moreover, there were significantly higher expression levels for HN-NDV in cancer spheroids (AMJ13 and MCF7) when compared to normal infected cells (hATMSCs) (Figure 7C). These results confirm that new NDV AMHA1 could infect and replicate efficiently in cancer spheroids only, with no significant replication in normal spheroids. Figure 7D summarizes the NDV replication cycle inside the infected cancer cells, where the NDV inserts its HN protein with fusion protein in the cell surface to help for a new virion assembly, and the budding and staining with HN-specific mAbs after 24 h of infection.

## Discussion

To the best of our knowledge, this is the first study testing the oncolytic efficiency of NDV virotherapy in a 3D model of breast cancer and normal mesenchymal stem cells. In cocultures with normal stem cells, we used the following breast cancer cell lines: AMJ13, estrogen, progesterone receptor–negative, and MCF7, progesterone receptor–positive breast cancer cell lines. We used two different breast cancer cell lines to cover both important types of breast cancer: hormone dependent and hormone independent.

**FIGURE 8:**
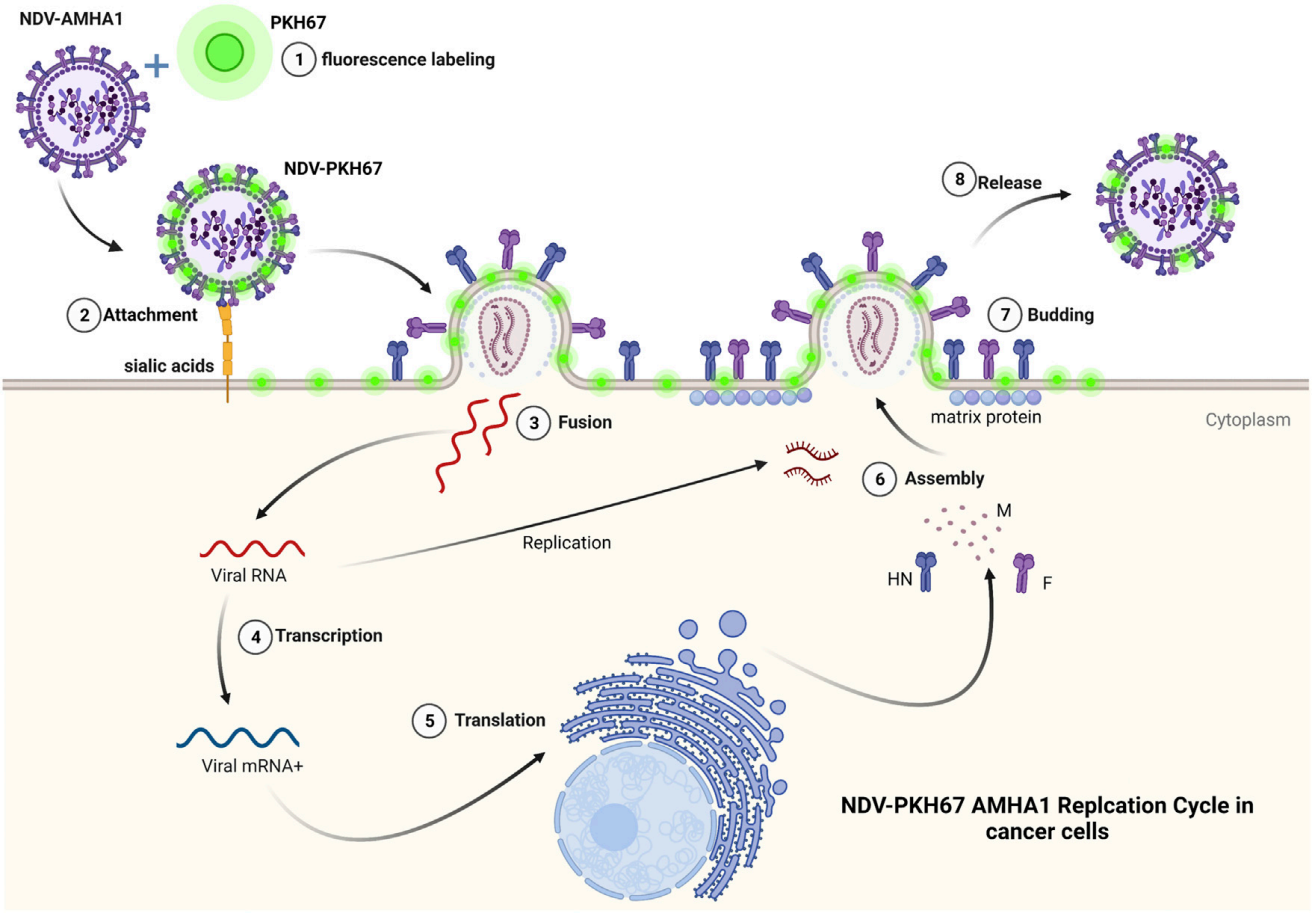
PKH67 fluorescent dye used to label the phospholipid membrane of NDV particles and used them as a tracking system to evaluate NDV oncolytic activity in cancer cells.

We used PKH67 fluorescent dye to label the phospholipid membrane of NDV particles and used them as a tracking system to evaluate NDV oncolytic activity in breast cancer–normal stem cell spheroids (Figure 8). This model represents a good model for oncolytic virotherapy to simulate *in vivo* experiments as Mandel et al. (2013) had described breast cancer cells cocultured with stem cells seemed to facilitate tumor cell expansion through interaction that promoted extracellular matrix structures of a tumor microenvironment, which protects breast cancer cells from chemotherapy treatment. One of the major obstacles to cancer virotherapy is the stromal and extracellular barriers those prevent viruses from dissemination inside the tumor mass (Howells et al., 2017). In the current study, we have reported the initial characterization of the 3D coculture sphere morphology and growth patterns in the scaffold panel, which successfully generated big spheroids of different sizes and shapes. It has been shown that 3D spheres are more like *in vivo* circumstances as nutrient, growth factors, and drug exposure are also heterogeneous, with cells on the outside edge of the spheroid being more exposed than cells in the inner core, thus resembling real tumors *in vivo*. Furthermore, malignant cells are grown on their microenvironment with normal cell signals for survival, growth as spheres aggregated to clusters, and metastasis (Aggarwal et al., 2012).

The promising finding of our work was that a simple technique of PKH67 fluorescent dye was used to label our NDV AMHA1 strain successfully to track virus entry, infectivity, and antitumor activity in our novel 3D coculture model. Another group used NDV expressing green fluorescent protein rFMW/GFP as a valuable method to track the efficiency of their strain NDV/FMW in killing cancer cells and determine the oncolytic mechanisms of NDV in inducing cell death (Jiang et al., 2018).

One possible way to investigate virus penetration of sphere cells is to label viruses with florescent dye and track the event of entry and vesicular trafficking to examine the virus’s penetrance as compared to controls. Since viruses are believed to bind to multiple receptor molecules, single-particle detection may also be used to study the effects of multiple receptor molecules (Ewers and Schelhaas, 2012). The Fluorescent Cell Linker PKH67 uses proprietary membrane labeling technology to stably incorporate a green fluorescent dye with long aliphatic tails (PKH67) into lipid regions of the cell membrane (Wallace et al., 2008). PKH67 is well suited for cytotoxicity assays that use propidium iodide or 7-aminoactinomycin D as viability probes (Zaritskaya et al., 2009; Awan et al., 2010; Tario et al., 2011). The PKH-based adsorption assay, besides its simplicity, is more rapid; after fusing NDV with the cell membrane, either receptor-mediated endocytosis of entire virus particles or constitutive pinocytosis of viral envelope sections might cause this. In the host cells, lateral migration of the dye molecules is expected to occur when the viral membranes fuse with the cell membrane (Balogh et al., 2011). PKH67 is often used for proliferation monitoring based on dye dilution (Barth et al., 2010; Rong et al., 2015), which includes the estimation of antigen-specific precursor frequencies (Schwaab et al., 2007), as well as for *in vivo* cell trafficking studies (Ledgerwood et al., 2008).

The labeled virus, oncolytic PKH67-NDV, replicated efficiently in breast cancer–hATMSCs spheroids. Furthermore, the PKH67-NDV AMHA1 strain showed significant oncolytic activity in breast cancer–hATMSCs 3D cultures as it inhibited sphere growth, as we can see a reduction in sphere size and number. PKH67-NDV inhibited the 3D growth potential of AMJ13 and MCF7 cells through lysing the spheres and other death mechanisms. Previously, we indicated that NDV AMHA1 induces both intrinsic and extrinsic apoptosis cell death involving caspase-dependent and -independent pathways (Mohammed et al., 2019). In the current study, PKH67-NDV induced lysis of breast cancer coculture 3D spheres as observed inside the cancer cell cytoplasm, which is fluorescent green, indicating virus presence that induces cancer cell death in response to PKH67-NDV infection. Our studies found that the NDV AMHA1 strain inhibits the glycolysis pathway in breast cancer cells by interfering with glycolysis enzymes such as hexokinase and GAPDH (Al-Shammari et al., 2019; Al-Ziaydi et al., 2020a). It has been reported that NDV can replicate in lung cancer spheroids and induce cell death in lung cancer stem cells (Hu et al., 2015).

We conducted a 2D culture and coculture assay to confirm the selectivity of NDV to infect cancer cells more than normal stem cells. The results show a significant increase in the high intensity of green fluorescence in cancer cells alone and in cocultures of cancer and normal cells treated with PKH-67 linker–labeled NDV as compared to the control coculture (PKH-67 linker without NDV) and labeled NDV–infected normal hATMSCs. The normal cells showed mild staining, reflecting some viral entry, but there was a huge difference when compared to that of cancer cells regarding the staining intensity, reflecting higher infectivity in cancer cells.

Moreover, the coculture system showed two populations of cells, first with a high-intensity green fluorescence and second, with a low intensity, in a very clear pattern, in both cancer cell lines tested: AMJ13 and MCF7. The high-intensity cells are the cancer cells, and the low-intensity ones are the normal cells. It has been found that MSCs can be used to carry oncolytic NDV to cancer cells such as glioma (Kazimirsky et al., 2016).

We established a 3D sphere system for cancer and normal cells separately to analyze the HN-NDV protein expression level by IF assay to confirm viral infectivity and replication inside cancer cells but not inside normal cells. The HN-NDV protein showed significant expression levels in the cancer spheroids for both cell lines, AMJ13 and MCF7, while there were no significant expression levels in the normal cells. These results prove that HN protein is expressed in infected cancer spheroids and has no expression in normal spheroids, confirming NDV selectivity in replication. These results confirmed the 2D experiment for halted virus replication in normal cells but efficient replication inside cancer cells.

The NDV genome encodes many proteins, such as NDV HN (hemagglutinin–neuraminidase); NDV HN is a glycosylated component of the external envelope responsible for NDV binding to host cells. Moreover, NDV HN binds to a receptor on target cells, causing an increase in the production of death receptors (DRs) and other proteins in the host cell (Chen et al., 2001).

After the virus and cell surface membranes have been bound, the NDV F protein regulates how they merge. The HN and F proteins promote infecting neighboring cells, contributing to NDV pathogenicity and virulence (Brown et al., 2021). HN protein contributes significantly to the replication and pathogenicity characteristics of NDV (Jin et al., 2016). NDV glycoprotein HN is synthesized by the rough endoplasmic reticulum and transported *via* the smooth intracellular membranes to the cell surface plasma membrane and into new NDV progeny (Nagai et al., 1976). Therefore, HN detection in the infected cell only confirms virus replication in cancer cells, as our immunofluorescence assay results have shown.

Our previous work revealed that classical chemotherapeutics, as well as different types of phytotherapeutics, enhance NDV AMHA1 oncolytic activity against breast cancer cells both *in vitro* and *in vivo*, as well as against many other cancer cell types (Al-Shammari, et al., 2016; Al-Shammari et al., 2020b). These data suggest promising strategies of NDV AMHA1 combination with chemotherapy or novel antitumor treatments that can increase oncolytic activity in breast cancer coculture 3D spheres to overcome the expected resistance of different types of cancer. NDV oncolytic activity involves the induction of a specific immune response against the infected cancer cells by inserting its antigens in the cell plasma membrane, as documented by our study in detecting HN protein on the infected cancer cell surface. This antigenic modification to the tumor cell surface can increase its recognition by the immune system leading to a specific immune response that helps in tumor elimination.

## Conclusion

We introduced the first use of breast cancer–normal stem cells 3D cocultures *in vitro* model to evaluate NDV antitumor activity. The 3D coculture model better resembles the *in vivo* environment *in vitro* regarding the barriers preventing oncolytic virus spread inside the tumor mass. The results showed that oncolytic NDV could destroy breast cancer 3D spheres while sparing the normal cells. Such results are encouraging for the clinical trials of oncolytic NDV AMHA1 as breast cancer therapy.

## Data Availability

The original contributions presented in the study are included in the article/supplementary material, and further inquiries can be directed to the corresponding author.
